# PA applicant U.S. citizenship status and likelihood of program matriculation

**DOI:** 10.1186/s12909-022-03947-x

**Published:** 2022-12-20

**Authors:** Mary Showstark, Michael Bessette, Carey L. Barry, Shahpar Najmabadi, Joanne Rolls, Catherine Hamilton, Virginia L. Valentin, Alicia Quella, Trenton Honda

**Affiliations:** 1 Yale University Physician Assistant Online Program, 100 Church Street South, Suite A230, New Haven, CT 06519 USA; 2 Department of Medical Sciences, Northeastern, University, 360 Huntington Ave, Boston, MA USA; 3 Department of Family and Preventive Medicine, University of Utah, 375 Chipeta Way, Ste. A, Salt Lake City, UT 84108 USA; 4 Retired, Bouve College of Health Sciences, Northeastern University, 360 Huntington Ave, Boston, MA USA; 5 Department of Physician Assistant Studies, University of Kentucky, 920 S. Limestone Suite 205, Lexington, KY 40536 USA; 6 People’s Center Clinic and Services, Minneapolis, MN USA; 7 School of Clinical and Rehabilitation Sciences, Northeastern University, 360 Huntington Ave, Boston, MA USA

**Keywords:** Physician assistant/associate, PA, Medical education, Matriculation, US Citizenship

## Abstract

**Background:**

Barriers to matriculation into Physician Assistant (PA) programs and entry into the PA profession have disproportionate impact on historically marginalized groups. This study evaluates if U.S. citizenship status is associated with likelihood of matriculation in PA Programs.

**Methods:**

Data from five Centralized Applicant Services for Physician Assistants (CASPA) admissions cycles (2012-2021) was evaluated cross-sectionally for the primary outcome of binary matriculation status (yes/no). Bivariate and multivariate logistic regression was utilized to investigate associations between self-identified U.S. citizenship status and likelihood of PA program matriculation. Models controlled for important potential confounders, including age, gender, race/ethnicity, non-native English speaker, patient care experience hours, total undergraduate grade point average (GPA), and number of applications submitted to various programs.

**Results:**

Non-U.S. citizen status was statistically associated with persistent lower likelihood of PA program matriculation compared to U.S. citizenship. Odds of matriculation were 41% [OR 0.59 (95% CI: 0.51, 0.68; *p* <.001)] to 51% [OR 0.49 (95% CI: 0.41, 0.58; *p* <.001)] lower in unadjusted models. Odds were 32% [OR 0.68 (95% CI: 0.56, 0.83; *p* <.001)] to 42% OR 0.58 (95% CI: 0.48, 0.71; *p* <.001) lower when adjusting for important covariates. The lowest likelihood occurred in 2012-2013 with 51% lower odds of matriculation and in 2016-2017 with 42% lower odds when accounting for important covariates.

**Discussion:**

PA programs are charged with improving diversity of clinically practicing PAs to improve health outcomes and better reflect patient populations. This analysis shows that non-U.S. citizenship may be a barrier to PA school acceptance. PA schools should raise awareness and create means and accessibility for admissions for this underrepresented group.

## Introduction

Admissions to U.S. Physician Assistant (PA) programs is an increasingly competitive and rigorous process, with over 21,000 applicants vying for just over 8,000 seats in 2020 [1]. Complicating the process is that each of the 293 colleges and universities that house PA programs have different application requirements [2, 3]. While a standardized application system known as the Central Application Services for Physician Assistants/Associates (CASPA™) helps applicants navigate these requirements, additional matriculation requirements or proscriptions often are listed on individual program websites, adding to the time, cost, and complexity associated with applying [4]. These barriers to admission likely differentially impact the likelihood of successful matriculation in historically marginalized and/or otherwise vulnerable groups. For example, a recent study found that underrepresented minority (URM) and older applicants had lower matriculation odds compared to white and younger individuals, but that these associations became not significantly different from the null when controlling for academic achievement and GRE status [5]. A subsequent study found that the number of programs to which an applicant applied was a strong predictor of likelihood of matriculation, and that associations differed by applicant race and ethnicity [6]. While studies increasingly document the barriers faced by some demographic groups in PA program matriculation, other demographic groups remain unstudied. For example, it is presently unknown whether applicant US citizenship status impacts matriculation likelihood. Non-US citizen applicants often face significantly complex processes in securing the legal right to reside in the US for their education, the funding for their tuition and living expenses specifically, the ability to work whilst a student is often limited or nonexistent. Additionally, these students may also face additional fees, which may include supplemental applications, higher tuition rates, and for many international applicants, the Test of English as a Foreign Language (TOEFL), which is a standardized assessment of an international student’s proficiency in the English language [7]. Additionally, not all US PA programs permit non-US citizens to apply, which limits the pool of potential universities for these groups. According to one website, in 2019 only 67% of PA programs accepted applications from international applicants. Ostensibly this is for the same reason that international applicants are limited in their ability to gain admission into medical schools in the US, which is a function of the lack of federal tuition funding, student loan, and/or scholarships for this group. Taken together these are significant and substantial barriers which may impact non-US citizens from matriculating at US PA programs [8–11].

While all the aforementioned barriers exist for non-US citizen applicants, there is likely significant variability determined in large part by an individual applicant’s immigration status. PA program applicants come from many different citizenship statuses and backgrounds, including: U.S. Citizen, Permanent Resident, and Temporary Resident. In the U.S. one can, be born into citizenship or one can naturalize [12]. Naturalization is a process in which a person above the age of 18 becomes a citizen after being a permanent resident for at least 5 years (or via marriage for 3 years) [13, 14]. A Permanent Resident is a category of non-US Citizen residency status that grants an individual the right to legally live, attend school, and work in the U.S, through provision of a ‘green card’ [15]. International students coming to study in the US usually utilize a temporary resident visa mechanism, which for PA schools is commonly the F1 visa [16]. There are also people who gain resident status as refugees or asylum seekers [17] (See Table 1). Others may have been born or brought into the U.S. at a young age; however, their parents may have been staying in the country in an undocumented capacity, and these children are referred to as the ‘dreamers.’ These children, some of whom are now adults, are protected under the Deferred Action for Childhood Arrivals (DACA) act which to protects them from deportation and gives them a work permit renewable every two years [18].

**Table 1:** Terminology and Definition with Example [17, 22–25]

Terminology	Definition with Example
U.S. Citizen	A person who is born into the U.S. or who has become naturalized after 3-5 years as a permanent resident
Naturalization	A process in which a person above the age of 18 becomes a citizen after being a permanent resident for at least 5 years (or via marriage for 3 years)
Permanent Residents	Allowed to live and work in the U.S.
Green Card/Permanent Resident Card/lawful permanent resident alien	Allows a person to be a permanent resident in the United States
Refugee	A person who has fled their own country because they are at risk of serious human rights violations and persecution. The risks to their safety and life were so great that they felt they had no choice but to leave and seek safety outside their country because their own government cannot or will not protect them from those dangers. Refugees have a right to international protection.
Asylum seeker	Similar to the definition of refugee, but who hasn’t yet been legally recognized as a refugee and is waiting to receive a decision on their asylum claim. Seeking asylum is a human right.
Resident	A person who is legally working or living in a country
Conditional Residents	Married less than two years before receiving green card
Undocumented	People who are in the country illegally or without permission; unable to work or obtain driver’s license
Deferred Action for Childhood Arrivals (DACA)	Individuals protected under the Deferred Action for Childhood Arrivals (DACA) act which to protects them from deportation and gives them a work permit renewable every two years.
VISA	Allows a person to stay in the country for a set duration •tourist (B-2 visa) •students (F-1 visa) •cultural and exchange (J-1 visa) •business visitors or tourists (B1/B2 visas) •fiancées (K-1 visa) •individuals granted temporary protected status •many others * https://travel.state.gov/content/travel/en/us-visas/visa-information-resources/all-visa-categories.html

Differential access to PA programs for non-US Citizens may have important implications for diversity in the PA profession. The Accreditation Review on the Education of the Physician Assistant (ARC-PA), the US accreditor for PA programs, states that each program should strive to increase diversity, equity, and inclusion, to care for a varied patient population [19, 20]. According to the Office of Immigration Statistics within the Department of Homeland Security over 38% of legal permanent residents in the US in 2021 were from Latin America and 32% were from Asia. As such, it is likely that policies which systematically disadvantage US permanent residents detrimentally impact diversity in US PA programs.

While PA students may apply with various citizenship statuses, it is unknown whether, and to what extent non-US citizenship status impacts access to PA education for this group. This differential access may result from several sources. Non-US Citizens may struggle to find support when entering a PA program, including confusion regarding immigration and visa requirements, language and cultural barriers, and access to financial resources. What is more, the impact of these potential barriers may have changed over time as immigration and visa policies have changed or been updated, DACA is threatened by state legal actions, and as universities increase or decrease the resources to support these student groups [21]. While some barriers may be lessened for permanent residents versus international applicants applying through a temporary resident visa mechanism (i.e., the ability to apply for and receive federal financial aid) others may not. The impact of US citizenship on admissions to PA programs is not well understood. The purpose of this study is to examine the association between applicant citizenship status and likelihood of US PA program matriculation from 2012-2020.

## Method and materials

### Data source and setting

To investigate the effect of applicants’ citizenship status on likelihood of matriculation to a U.S. physician assistant program, we utilized data from five Centralized Application Service for Physician Assistants (CASPA) admission cycles, including 2012-2013, 2014-2015, 2016-2017, 2018-2019 and 2020-2021. Over 90% of the accredited national PA programs use CASPA for student recruitment [1]. Participants included all non-duplicate applicants to the respective admission cycle.

### Ethics approval

Access to the de-identified data was provided by the Physician Assistant Education Association (PAEA). At the time of submission, all applicants signed an agreement releasing their CASPA data to PAEA with permission to analyze and report deidentified data in aggregate. The study was determined exempt by the Northeastern University Institutional Review board.

### Outcome and covariates

Our primary outcome of interest was matriculation status (Yes/No) to a U.S. PA program. Applicants were considered matriculated to a PA program if any program indicated ‘matriculated’. All other answers, (i.e., ‘offer made’, ‘wait listed’, ‘deferred’) were considered non-matriculated. Applicants could apply for more than one program, however, only one matriculation was possible.

Our primary predictor of interest was self-reported citizenship status. The binary variable of citizenship (Yes/No) included ‘U.S. Citizen’ applicants versus all other mentioned categories, (i.e., 'Non-Resident', 'None', 'Temporary Resident', 'Temporary U.S. Resident', 'Permanent Resident', and 'Permanent U.S. Resident').

In addition to citizenship status, models were adjusted for potential confounders which have previously been found to be associated with matriculation status, including: age at time of application (continuous), race/ethnicity (defined as 5 conditional categories of Black/African American, American Indian/Alaska Native or Native Hawaiian/Pacific Islanders, Asian, Hispanic and non-Hispanic White), whether the student identified as a non-native English speaker (English as second language, ESL), patient care experience hours (9-level categorical variable based on self-reported prior experience), total undergraduate grade point average (GPA) with (7-level categorical variable), and number of applications submitted to various programs by a given applicant (3-level categorical variable, adapted from a recent study)1, and applicant gender (3-level categorical variable: male, female, decline to state) [6].

### Statistical analysis

We utilized logistic regression to investigate association between applicant citizenship status and likelihood of program matriculation in both bivariate and multivariable regression models controlling for: age, gender, race/ethnicity, patient care experience, ESL status, total undergraduate GPA, and number of applications submitted to various programs. To ensure our model specifications were not unduly influencing our results, in sensitivity analyses we explored 1) whether a less restrictive outcome definition meaningfully impacted the associations observed by including 'Permanent Resident', and 'Permanent U.S. Resident' designations in the “US Citizen” category, and 2) the associations between permanent residents to US citizens and non-citizen-non-permanent residents to US citizens.

## Result

Table 2 summarizes the demographic characteristics of our analytical cohorts. Overall, the number of CASPA admission cycle applicants increased over time from 19,723 in 2012-2013 to 30,123 applicants in the 2020-2021 cycle. Matriculation rates varied over time, with the highest matriculation rate in the 2018-2019 cycle (38.02%) and lowest matriculation rate in the 2020-2021 cycle (27.64%). Overall, matriculants were younger than non-matriculants, and there was a decreasing trend in age of both matriculants and non-matriculant over the selected admission cycles, with the youngest matriculants in admission cycle 2020-2021 (24.42 years +/- 4.22).

**Table 2:** Demographic characteristics of physician assistant applicants for the selected CASPA cycle years by matriculation status [mean (SD) or count (%)]

	**Non-matriculated**					**Matriculated**				
	**2012**	**2014**	**2016**	**2018**	**2020**	**2012**	**2014**	**2016**	**2018**	**2020**
	13543 (68.67)	15189 (67.40)	17979 (67.32)	16608 (61.98)	21798 (72.36)	6180 (31.33)	7348 (32.60)	8729 (32.68)	10187 (38.02)	8325 (27.64)
**Age at app submission**										
** Mean (SD)**	26.64 (6.62)	26.47 (6.37)	26.40 (6.05)	26.42 (5.93)	26.15 (5.90)	25.31 (5.46)	25.02 (5.06)	24.85 (4.88)	24.56 (4.39)	24.42 (4.22)
** Median**	24	24	24	24	24	24	23	23	23	23
**Sex**										
** Female**	9594 (70.84)	10759 (70.83)	12756 (70.95)	11884 (71.56)	16215 (74.39)	4474 (72.39)	5322 (72.43)	6411 (73.44)	7617 (74.77)	6430 (77.24)
** Male**	3924 (28.97)	4386 (28.88)	5219 (29.03)	4700 (28.30)	5552 (25.47)	1699 (27.49)	2018 (27.46)	2317 (26.54)	2565 (25.18)	1885 (22.64)
** Declined to state**	25 (0.18)	25 (0.16)	4 (0.02)	24 (0.14)	31 (0.14)	7 (0.11)	6 (0.08)	1 (0.01)	5 (0.05)	10 (0.12)
**Race/Ethnicity**										
** NL White**	8419 (62.16)	9303 (61.25)	10041 (55.85)	9355 (56.33)	12205 (55.99)	4656 (75.34)	5342 (72.70)	5869 (67.24)	6995 (68.67)	5692 (68.37)
** Black/African American**	1244 (9.19)	1335 (8.79)	1600 (8.90)	1839 (11.07)	2454 (11.26)	222 (3.59)	286 (3.89)	355 (4.07)	447 (4.39)	434 (5.21)
** Hispanic**	1244 (9.19)	1527 (10.05)	1919 (10.67)	2077 (12.51)	3002 (13.77)	433 (7.01)	558 (7.59)	778 (8.91)	960 (9.42)	849 (10.20)
** Asian**	1435 (10.60)	1620 (10.67)	1904 (10.59)	1821 (10.96)	2558 (11.74)	434 (7.02)	602 (8.19)	719 (8.24)	985 (9.67)	854 (10.26)
** American Indian/Pacific Islander**	748 (5.52)	797 (5.25)	1023 (5.69)	915 (5.51)	1094 (5.02)	219 (3.54)	260 (3.54)	314 (3.60)	433 (4.25)	336 (4.04)
**Citizenship status**										
** US citizen**	12809 (94.58)	14492 (95.41)	17208 (95.71)	15907 (95.78)	20970 (96.20)	6011 (97.27)	7155 (97.37)	8505 (97.43)	9953 (97.70)	8144 (97.83)
** Not US citizen**	726 (5.36)	679 (4.47)	771 (4.29)	701 (4.22)	828 (3.80)	167 (2.70)	191 (2.60)	224 (2.57)	234 (2.30)	181 (2.17)
** • ESL/Yes**	323 (44.49)	313 (46.10)	281 (36.45)	289 (41.23)	361 (43.60)	78 (46.71)	88 (46.07)	85 (37.95)	102 (43.59)	73 (40.33)
** • ESL/No**	403 (55.51)	366 (53.90)	490 (63.55)	412 (58.77)	467 (56.40)	89 (53.29)	103 (53.93)	139 (62.05)	132 (56.41)	108 (59.67)

*CASPA* Centralized application service for physician assistants, *SD* Standard deviation, *ESL* English as second language

Across all analytical cohorts, the majority of applicants were female with increasing trend over time for both matriculants and non-matriculants, and a trend of higher female matriculants compared to male matriculation was also observed.

Over the study timeframe, the proportion of matriculants identifying as non-Hispanic White decreased monotonically over time (75.34% in 2012-2013 versus 68.37% in 2020-2021). Correspondingly, applicants identifying in all other racial/ethnic groups saw increases in the proportion of matriculation. For example, in 2012-2013, 3.59% of matriculated students identified as Black/African American while in 2020-2021 this was 5.21%. While the proportion of underrepresented minority matriculants increased over time, the proportion of total Black and other racial/ethnic minority applicants to matriculate was still well below that of non-Hispanic White applicants. In 2020-2021, there were 2.16 Black applicants not matriculating for every 1 Black applicant who did matriculate (2.16:1), while for non-Hispanic White applicants this ratio was 0.82:1.

The proportion of applicant cohorts self-identifying as non-US citizens was small across our whole study timeframe, with the smallest proportions of matriculants (2.17%) and non-matriculants (3.8%) identifying as non-US citizens observed in 2020-2021. Overall, among non-citizen applicants, over 50% specified English as their native language.

Table 3 shows the results of our bivariate and multivariate logistic regression models. In unadjusted models, non-U.S. citizen status was associated with a significantly lower likelihood of matriculation to a PA program across all years of our study, with the strongest associations observed for 2012-2013, for which non-US citizen status was associated with 51% lower odds of matriculation (95% CI: 32%, 59%) compared to US citizens. In adjusted multivariable models, associations were somewhat attenuated, but remained inverse and significant across all years, with the strongest associations observed for 2016 (OR: 0.58, 95% CI: 0.48, 0.71). Our sensitivity analysis examining a different definition of citizen status (included permanent resident and permanent US resident) did not importantly differ from our main findings (Table 4) In our sensitivity models comparing permanent residents to US citizens and non-citizen-non-permanent residents to US citizens (Table 5), we did not identify important differences in likelihood of matriculation between these groups relative to US citizens.

**Table 3:** Associations between applicant non-US citizen status and PA program matriculation by year

	**Unadjusted**				**Adjusted** ^a^			
**Year**	**Estimate** **OR**	**95% CI**		**P**	**Estimate** **OR**	**95% CI**		**P**
**2012**	0.49	0.41	0.58	<.001	0.62	0.50	0.77	<.001
**2014**	0.57	0.48	0.67	<.001	0.61	0.50	0.75	<.001
**2016**	0.59	0.51	0.68	<.001	0.58	0.48	0.71	<.001
**2018**	0.53	0.46	0.62	<.0001	0.62	0.51	0.75	<.001
**2020**	0.56	0.48	0.66	<.0001	0.68	0.56	0.83	<.001

*CASPA* Centralized application service for physician assistants, *OR* Odd ratio, *CI* Confidence interval

^a^Adjusted for age at application submission, English as second language, binary gender, race/ethnicity, application number, hours of patient experience, and cumulative undergraduate total grade point average

**Table 4:** Sensitivity Analysis ^a^ Associations between non-US citizen status (excluding “Permanent Resident” and “Permanent US Resident) and PA program matriculation by year

	**Unadjusted**				**Adjusted** ^b^			
**Year**	**Estimate** **OR**	**95% CI**		**P**	**Estimate** **OR**	**95% CI**		**P**
**2012**	0.49	0.41	0.58	<.001	0.62	0.50	0.77	<.0001
**2014**	0.52	0.37	0.74	<.001	0.58	0.39	0.88	0.001
**2016**	0.51	0.37	0.69	<.001	0.56	0.38	0.81	<.001
**2018**	0.54	0.42	0.71	<.001	0.75	0.54	1.04	0.088
**2020**	0.44	0.32	0.60	<.001	0.54	0.38	0.77	0.001

*CASPA* Centralized application service for physician assistants, *OR* Odd ratio, *CI* Confidence interval

^a^Considered ‘Permanent Resident’, ‘Permanent U.S. Resident’ same as US citizens

^b^Adjusted for age at application submission, English as second language, binary gender, race/ethnicity, application number, hours of patient experience, and cumulative undergraduate total grade point average

**Table 5:** Sensitivity Analysis Associations between non-US citizen status (comparing permanent residents to US citizens and non-permanent residents to US citizens) and PA program matriculation by year

	**Unadjusted**				**Adjusted** ^a^			
**Year**	**Estimate** **OR**	**95% CI**		**P**	**Estimate** **OR**	**95% CI**		**P**
**2014**								
Non-Permanent Resident	0.51	0.36	0.73	0.0002	0.55	0.36	0.82	0.0037
Permanent Resident	0.59	0.49	0.71	<.0001	0.63	0.51	0.79	<.0001
**2016**								
Non-Permanent Resident	0.50	0.37	0.68	<.0001	0.53	0.37	0.77	0.0009
Permanent Resident	0.62	0.52	0.73	<.0001	0.60	0.48	0.75	<.0001
**2018**								
Non-Permanent Resident	0.54	0.41	0.70	<.0001	0.71	0.51	0.99	0.042
Permanent Resident	0.53	0.45	0.64	<.0001	0.59	0.47	0.74	<.0001
**2020**								
Non-Permanent Resident	0.44	0.32	0.60	<.0001	0.53	0.37	0.75	0.0004
Permanent Resident	0.63	0.52	0.76	<.0001	0.76	0.61	0.95	0.0161

*CASPA* Centralized application service for physician assistants, *OR* Odd ratio, *CI* Confidence interval

Adjusted for age at application submission, English as second language, binary gender, race/ethnicity, application number, hours of patient experience, and cumulative undergraduate total grade point average

## Discussion

Ours is the first study to show that non-U.S. citizen status is associated with persistent, sizeable, and significant decreases in likelihood of matriculation to U.S. physician assistant programs. This decreased likelihood of matriculation was consistent across the timeframe of our study and was only minimally attenuated in our fully adjusted statistical models accounting for ESL status, academic achievement, and patient care experience, and was largely unchanged when we examined different definitions of citizenship. In sensitivity analyses comparing non-permanent resident and permanent residents to U.S. Citizens, we found only nominal differences between these groups, suggesting that federal student aid is not the only driver of the differences we observe.

It has long been known that international medical students faced significant challenges in the U.S., and it is likely that many of the same barriers are driving our findings among non-U.S. citizen PA applicants. Some of this is due to a combination of logistical factors mainly stemming from the high cost of medical education in the United States and the lack of available funding or loan mechanisms for international students. In fact, many private medical schools will accept international students if and only if they are able to pay for the duration of their schooling in advance [9]. The impacts of these obstacles are large, with the American Association of Medical Colleges reporting the 2019 international student acceptance rate (17.2%) at less than half that of domestic students [26]. Surprisingly however, when we examined temporary resident (which includes most international F1 visa applicants) and permanent residents (which includes ‘green card’ holders, refugees, and asylum seekers) separately, the disparity in access persisted for permanent residents despite this latter group being explicitly eligible for financial aid [27, 28]. That the results were not importantly different between bivariate and multivariable models further indicates that this is unlikely to be due to confounding by ESL status, academic achievement, applicant demographics, and patient care experience. The reason for this residual decrement in likelihood of matriculation remains unclear.

The associations we observed were also remarkably preserved across time, with no obvious trend of increasing or decreasing association. There are a few possible reasons for the persistence of our findings. While many programs have worked to increase diversity, the obstacles in licensing and employment by international graduates has not changed. There have also not been noteworthy changes in the requirements for student visas or increases in funding for international students. There are also likely implicit bias factors which impact the admissions process, from those surrounding race and ethnicity to those regarding perspectives on undergraduate degrees obtained from outside of the United States [29, 30]. The complexity of the US immigration system is highlighted in the sensitivity analysis where it is likely that many on admissions committees do not know the difference between a permanent resident and say a conditional resident. Lastly, if these barriers are overcome it is likely that a lack of support, affinity, and guidance in the pre-matriculation phase could impact admission likelihood.

We believe that these findings have several important implications, particularly pertaining to diversity within the profession. As described above, over 38% of legal permanent residents in the US in 2021 were from Latin America and 32% were from Asia. As such, it is likely that policies which systematically disadvantage US permanent residents detrimentally impact diversity in US PA programs. PA schools may need to consider raising awareness and create means and accessibility for admissions for this underrepresented group.

Another important implication is the burgeoning nature of the PA profession on the international stage. As more countries develop and implement a PA model of care, the profession might benefit from a warmer approach to educating international students who will ultimately pursue a career in their home country or go abroad to practice as a PA. While currently, state licensing boards require that PAs graduate from an ARC-PA accredited program, and all such programs are located in US states or territories, the possibilities to go and work abroad include countries such as Canada, U.K., Germany, Netherlands, Ireland, and South Africa. These opportunities may be as a practicing PA, an educator, or with a non-profit organization. There are also opportunities for PAs to practice abroad with the government and military branches. A less austere approach to international applicants by US PA programs may help to further foster this international interest in, and respect for, the PA profession.

### Study strengths/limitations

Our study has several important limitations. First, while we had access to numerous years of national CASPA data, approximately 5% of US PA programs do not utilize the CASPA application system and as such our findings may not be generalizable to that group. Second, the CASPA data is largely dependent upon applicant self-report of data. As such, it is possible that we had exposure misclassification which could lead to information bias in the event that there is differential misclassification which could occur if, for example, non-citizen students were more likely to misclassify their citizenship status than US citizens. Additionally, as we capture matriculant and not acceptance data, it is possible that applicants were admitted, but chose not to matriculate after learning about the financial and logistical barriers they might face. This study also cannot take into account the admissions processes and policies that occur at each program including but not limited to the University level policies around acceptance of non-US citizens.

These weaknesses are counterbalanced by a number of important strengths. Our study presents the first robust analysis exploring the relationship of citizenship status and matriculation in PA programs in the United States. It is strengthened by the use of robust national datasets from CASPA, which provides access to high quality predictor, outcome, and confounder information.

## Conclusions

PA programs are charged with improving the diversity of clinically practicing PAs to improve population health outcomes and better reflect the patients they serve. This charge has been incorporated in ARC-PA accreditation standards and has been encouraged by the four U.S. national physician assistant organizations Our findings of lower likelihood of matriculation among non-US citizens, an important and diverse community, provides potential opportunities for PA programs and their sponsoring institutions to reconsider policies which may disadvantage these groups.

## Data Availability

The data that support the findings of this study are available from the Physician Assistant Education Association (PAEA), but restrictions apply to the availability of these data, which were used under license for the current study, and so are not publicly available. Data are however available from the authors upon reasonable request and with permission of PAEA.

## References

[1] Chitwood R, Yuen C. By the Numbers: Program Report 35.; 2020. doi:10.17538/PR35.2020

[2] CASPA for PA Programs | PAEA. Accessed August 18, 2022. https://paeaonline.org/resources/member-resources/caspa

[3] Accredited Programs – ARC-PA. Accessed September 19, 2022. http://www.arc-pa.org/accreditation/accredited-programs/

[4] The PA Pipeline to Practice. Accessed September 19, 2022. https://paeaonline.org/wp-content/uploads/2020/10/paea-presentation-caspa-20200106.pdf

[5] Yuen CX, Honda TJ (2019). Predicting Physician Assistant Program Matriculation Among Diverse Applicants. Acad Med.

[6] Honda T, Henry TD, Mandel ED, et al. Maximizing Black applicant matriculation in U.S. PA programs: associations between the number of submitted applications and likelihood of matriculation. BMC Med Educ. 2021;21(1):127. doi:10.1186/s12909-021-02563-5 10.1186/s12909-021-02563-5 PMC7901109 33622312

[7] Prepare for the TOEFL Essentials Test. Accessed September 19, 2022. https://www.ets.org/toefl/test-takers/essentials/prepare.html

[8] 165 US PA Programs That Accept International Students | The Physician Assistant Life. Accessed July 19, 2022. https://www.thepalife.com/us-pa-programs-that-accept-international-students/

[9] Obstacles Facing International Pre-medical Students - Biomedical Sciences Department - Missouri State. Accessed September 19, 2022. https://www.missouristate.edu/BMS/Undergraduate/obstacles.htm

[10] Datta J, Miller BM (2012). International students in United States’ medical schools: does the medical community know they exist?. Med Educ Online.

[11] Miller EJ HJ. International students and medical education: Available, options and obstacles. Advis. 24:(44 7.).

[12] Volume 12 - Citizenship and Naturalization | USCIS. Accessed September 19, 2022. https://www.uscis.gov/policy-manual/volume-12

[13] Citizenship and Naturalization | USCIS. Accessed September 19, 2022. https://www.uscis.gov/citizenship/learn-about-citizenship/citizenship-and-naturalization

[14] Chapter 3 - U.S. Citizens at Birth (INA 301 and 309) | USCIS. Accessed September 19, 2022. https://www.uscis.gov/policy-manual/volume-12-part-h-chapter-3

[15] Green Card | USCIS. Accessed September 19, 2022. https://www.uscis.gov/green-card

[16] Students and Employment | USCIS. Accessed September 19, 2022. https://www.uscis.gov/working-in-the-united-states/students-and-exchange-visitors/students-and-employment#:~:text=F-1 Student Visa,in a language training program

[17] GBlakeley. USCIS Welcomes Refugees and Asylees. Accessed September 19, 2022. https://www.uscis.gov/sites/default/files/document/brochures/USCIS_Welcomes_Refugees_and_Asylees.pdf

[18] Consideration of Deferred Action for Childhood Arrivals (DACA) | USCIS. Accessed September 19, 2022. https://www.uscis.gov/DACA

[19] Accreditation Standards for Physician Assistant Education ↖.; 2019. Accessed August 2, 2020. www.arc-pa.org

[20] Cuenca JP, Ganser K, Luck M, Smith NE, McCall TC (2022). Diversity in the Physician Assistant Pipeline: Experiences and Barriers in Admissions and PA School. J Physician Assist Educ.

[21] Additional Information: DACA Decision in State of Texas, et al., v. United States of America, et al., 1:18-CV-00068, (S.D. Texas July 16, 2021) (“Texas II”) | USCIS. Accessed September 19, 2022. https://www.uscis.gov/humanitarian/consideration-of-deferred-action-for-childhood-arrivals-daca/additional-information-daca-decision-in-state-of-texas-et-al-v-united-states-of-america-et-al-118-cv

[22] Directory of Visa Categories. Accessed September 19, 2022. https://travel.state.gov/content/travel/en/us-visas/visa-information-resources/all-visa-categories.html

[23] Refugees, Asylum-seekers and Migrants - Amnesty International. Accessed August 20, 2022. https://www.amnesty.org/en/what-we-do/refugees-asylum-seekers-and-migrants/

[24] Baugh R. Annual Flow Report September 2020 - Refugees and Asylees: 2019.; 2020. Accessed April 12, 2022. https://www.dhs.gov/sites/default/files/publications/immigration-statistics/yearbook/2019/refugee_and_asylee_2019.pdf

[25] Baugh R. Fiscal Year 2020 Refugees and Asylees Annual Flow Report OFFICE OF IMMIGRATION STATISTICS.; 2022. Accessed April 12, 2022. https://www.dhs.gov/sites/default/files/2022-03/22_0308_plcy_refugees_and_asylees_fy2020_1.pdf

[26] Table A-12 : Applicants , First-Time Applicants , Acceptees , and Matriculants to U . S . MD-Granting Medical Schools by Race / Ethnicity ( Alone ), 2018-2019 through 2021-2022 Table A-12 : Applicants , First-Time Applicants , Acceptees , and Matriculants. Published online 2022:2018-2019.

[27] Eligibility for Non-U.S. Citizens | Federal Student Aid. Accessed September 19, 2022. https://studentaid.gov/understand-aid/eligibility/requirements/non-us-citizens

[28] Association of American Medical Colleges (AAMC). Applying to Medical School as an International Applicant. AAMC.

[29] Azan Zahir Virji M. Association of American Medical Colleges (AAMC). As a non-U.S. citizen, I faced hurdles applying to U.S. medical schools. Now that I’ve made it, I want to help others like me. Accessed September 6th, 2022. AAMC. Published online 2021. https://www.aamc.org/news-insights/non-us-citizen-i-faced-hurdles-applying-us-medical-schools-now-i-ve-made-it-i-want-help-others-me

[30] Capers Q, Clinchot D, McDougle L, Greenwald AG (2017). Implicit Racial Bias in Medical School Admissions. Acad Med.

